# Assessing the impacts of having a child with achondroplasia on parent well-being

**DOI:** 10.1007/s11136-020-02594-3

**Published:** 2020-08-16

**Authors:** Kathryn M. Pfeiffer, Meryl Brod, Alden Smith, Jill Gianettoni, Dorthe Viuff, Sho Ota, R. Will Charlton

**Affiliations:** 1 The Brod Group, 219 Julia Ave., Mill Valley, CA 94941 USA; 2 Ascendis Pharma, Inc, Palo Alto, CA USA; 3 Ascendis Pharma, A/S, Hellerup, Denmark

**Keywords:** Achondroplasia, Caregivers, Emotional well-being, Patient-reported outcome measures, Quality of life, Work

## Abstract

**Purpose:**

This study’s purpose was to develop a better understanding of the experiences of parents of children with achondroplasia and to provide qualitative evidence to support the development of a patient-reported outcome (PRO) measure of parent impacts.

**Methods:**

Concept elicitation (CE) individual/focus group interviews were conducted with parents of children aged 2 to < 12 years with achondroplasia in the United States and Spain. The qualitative analysis informed the PRO measure development. Cognitive debriefing (CD) interviews were conducted to ensure parent understanding and item relevance.

**Results:**

Thirty-six parents participated in individual/focus group CE interviews. The analysis identified parent impacts in four domains, including caretaking responsibilities, emotional well-being, family, and work, and results informed the development of the Achondroplasia Parent Experience Measure (APEM). Caretaking responsibilities included managing child’s medical care (92%), helping child with self-care (67%), advocating for child (64%), assisting child (56%), and observing/monitoring child (e.g., to ensure safety; 47%). Impacts on parents’ emotional well-being included worry about the future (75%), worry about child’s physical health (67%), safety concerns (50%), feeling stressed/overwhelmed (44%), and worry about child’s social relationships (42%). Impacts on family and work included family strain (56%), limiting/adapting family activities (42%), and missed work time (50%). CD interviews with an additional 16 parents of children with achondroplasia confirmed understanding and item relevance.

**Conclusion:**

The results improve our understanding of the experiences of parents of children with achondroplasia and provide qualitative evidence to support the content validity of the APEM. A psychometric study is needed to validate the measure.

## Introduction

Achondroplasia is the most common form of dwarfism, affecting more than 250,000 people worldwide with an incidence of between 1/10,000 and 1/30,000 live births [1]. The condition results from a mutation in the *FGFR3* gene, which affects many tissues, especially bone and cartilage [1, 2]. Clinical indicators of achondroplasia include short stature, disproportional shortening of the legs and arms, macrocephaly with frontal bossing, midface hypoplasia, small chest size, abnormal curvature of the spine, trident-shaped hands, joint hypermobility, and tibial bowing [3].

Frequent complications of achondroplasia in childhood include recurrent ear infections, sleep apnea, hearing loss, teeth crowding/misalignment, and speech delay [4, 5]. Pain in the back, legs, and joints, respiratory issues, spinal stenosis resulting in neurological symptoms (e.g., tingling/numbness in extremities), hydrocephalus, and foramen magnum compression may also occur [4, 5]. Common complications in adulthood include obstructive sleep apnea, spinal stenosis, overweight/obesity, chronic back/leg pain, hearing impairment, and respiratory problems [1, 3, 4, 6, 7]. Although there is currently no treatment for achondroplasia, clinical management guidelines are well established and new treatments are currently in development [1, 3, 5, 8–10].

While the clinical complications of achondroplasia in children are well known, less is known about the impacts of achondroplasia on children’s functioning and well-being. Infants and young children with achondroplasia frequently experience delayed developmental milestones in gross and fine motor skills, communication, and feeding [11, 12]. Delays in gross motor skills, communication, and self-feeding are particularly evident in children’s first two years of life [12]. Older children and adolescents with achondroplasia have been shown to experience lower quality of life in the areas of physical, emotional, social, and school functioning compared to a reference population [13].

In addition, little is known about how having a child with achondroplasia affects parents’ daily lives and well-being. Evidence suggests that parents of affected children may have more caretaking responsibilities, as children with achondroplasia have greater need for help with self-care and mobility compared to children developing typically [14]. Infants and young children must be monitored frequently due to risks of serious complications [8–10]. Children with achondroplasia often require minimal to moderate parent/caregiver assistance with self-care (e.g., toileting, dressing, bathing), and some need parent/caregiver supervision in social settings beyond the age of seven years [14]. Parents are also advised to arrange appropriate adaptations and accommodations for children at home and school to ensure children’s daily functioning, safety, and school participation [8]. Parents of adolescents may face additional challenges related to helping their children cope with increased stressors related to short stature and negative treatment from others due to achondroplasia, such as teasing, staring, and being stigmatized [15, 16].

Research has suggested that having a child with achondroplasia also has implications for parent well-being. Parents of children with achondroplasia have been shown to experience a range of emotions following their child’s diagnosis, including feelings of shock, sadness, worry, anger, and denial [17]. Parents of children and adolescents with achondroplasia also experienced significantly worse mental health on average, compared to a reference population, but no difference in physical health [13]. Additionally, parents’ general quality of life was positively and significantly correlated with parent-reported children’s quality of life [13].

Currently, there is limited understanding of the impacts of having a child with achondroplasia on parents’ well-being. The purpose of the study was to investigate how having a child aged 2 to < 12 years with achondroplasia affects parents, including impacts on caretaking responsibilities, physical health, emotional and social well-being, family life, and work. The study findings were intended to support the development of a parent-reported experience measure to assess the impacts of having a child with achondroplasia on parents’ daily life and well-being.

## Methods

The study used qualitative research methods in line with United States (US) Food and Drug Administration (FDA) guidelines for patient-reported outcome (PRO) measure development and best research practices [18–22]. Concept elicitation (CE) interviews were conducted with achondroplasia experts and parents of children with achondroplasia, aged 2 to < 12 years. The analysis of CE data, using an adapted grounded theory approach, informed the development of measure items and a preliminary theoretical model of the impacts of having a child with achondroplasia on parents. Cognitive debriefing (CD) interviews were conducted for the measure to assess clarity and relevance. The study was approved by an independent Institutional Review Board (IRB), Copernicus Group IRB, Research Triangle Park, North Carolina, US (Protocol numbers TBG1-18-117 and 20190578).

### Concept elicitation

A literature review and expert interviews were conducted to provide background information and clinical knowledge of achondroplasia and its impacts on affected children and their parents. Based on a semi-structured interview guide, expert interviews were conducted with clinical experts in the US and Spain and with one achondroplasia patient advocacy group leader in the US. Clinical expert eligibility criteria included (1) expertise in one or more fields related to achondroplasia; and (2) at least five years of clinical experience treating children with achondroplasia.

The literature review and expert interviews informed the development of a semi-structured interview guide to elicit parents’ experiences related to their child’s achondroplasia, including impacts on parents’ daily life, emotional and social well-being, family, and work. Inclusion criteria for parent participants included (1) adults aged 18 years or older; (2) able to read, write, and speak English (US) or Spanish (Spain); (3) parent of a child (aged 2 to < 12 years) diagnosed with achondroplasia; and (4) parent actively involved in child’s care. Exclusion criteria included having a cognitive impairment or medical/psychiatric condition that would affect one’s ability to participate in an interview/focus group. These interviews were part of a larger study, which included interviews with parents of children < 18 years of age with achondroplasia and interviews with older children/adolescents ages 9 to < 18 years with achondroplasia. Given the differences in the development and experiences of infants and young children < 2 years and adolescents with achondroplasia and their parents, these data were analyzed separately, and results will be reported in separate manuscripts.

A multi-pronged parent recruitment strategy was used: (1) achondroplasia advocacy organizations disseminated a study information sheet to members through email/social media; (2) clinical experts were asked to share study information with parents; (3) a professional market research organization recruited participants through social media; and (4) willing participants shared study information with other parents (“snowball” sampling). To ensure that a broad range of parent experiences was captured, recruiting targets were set for country, child age, and whether parent has achondroplasia. Given the developmental stages of children, as well as cultural variation in the perceptions of achondroplasia, parents of children of varying ages or residing in different countries may have differing perspectives and experiences related to their child’s achondroplasia. Additionally, parents who have achondroplasia themselves are likely to have different experiences compared to parents who do not.

Parents were required to verify their study eligibility before participating by answering a screening questionnaire through a brief telephone call with a recruiter. Informed consent was obtained from each participant prior to the interview, and participants received a modest honorarium.

Expert telephone interviews were conducted individually and lasted approximately 60 min. Expert interviews were conducted in English or Spanish, based on interviewee preference. Individual parent CE telephone interviews were conducted and lasted about 60 min. One parent focus group was held in-person in Spain and lasted about two hours. Individual/focus group interviews, conducted in English (US) and Spanish (Spain), were audio recorded and transcribed verbatim.

Interview/focus group transcripts were analyzed for conceptual themes using an iterative process and an adapted grounded theory approach [20]. Data analysis was conducted using Dedoose© [23]. The interview guides informed the development of a preliminary code list of impacts (concepts). Transcripts were coded in the chronological order in which they occurred. Concepts that emerged during the coding process were added to the code list, and transcripts coded previously were reviewed for new concepts. Concepts were organized into larger themes and sub-themes.

### Item generation

A two-day, in-person item generation meeting was held by the project team, including the interviewers and analyst. At the meeting, the team discussed the qualitative analysis and confirmed/modified codes as needed. Based on review of the analysis report, the team agreed on criteria for identifying impacts as major, and therefore considered for inclusion in the measure, and minor, which would not be considered. The team identified impacts that were important and relevant to parents of children with achondroplasia of differing ages and could potentially improve with a new treatment for children.

After identifying major and minor impacts on parents, the team created a preliminary theoretical model of the impacts of having a child with achondroplasia. The theoretical model’s purpose was to illustrate the key impact domains, to distinguish between temporally proximal and distal impacts, and to identify potential modifying factors. Informed by the qualitative analysis report, preliminary theoretical model, and major impacts/domains identified, a preliminary version of the Achondroplasia Parent Experience Measure (APEM) was developed to assess the impacts of having a child with achondroplasia aged 2 to < 12 years on parents. An item definition table was also created to define the conceptual meaning of instructions and items in the measure, using parents’ language when possible. Additionally, a translatability assessment was conducted to identify potential difficulties in translating the APEM.

### Cognitive debriefing interviews

Parent CD interviews were conducted to confirm that instructions and items were clear and relevant, that the structure/format was acceptable, and that the recall period was appropriate [21]. The CD interviews were conducted with an independent sample of parents of children with achondroplasia, aged 2 to < 12 years, who resided in the US and had not participated in CE interviews. Parent eligibility criteria matched the criteria for CE interviews described above. The multi-pronged recruitment approach used for the CE interviews was also used for the CD interviews. Parents received a modest honorarium for their participation.

Prior to the CD interviews, participants were emailed the APEM and were asked to complete it 24–48 h before their scheduled interview and to have the completed measure with them during the interview. Individual CD telephone interviews were conducted based on a structured interview guide, using a “think aloud” method and probing [21], and lasted approximately 90 min.

CD interviews were conducted in blocks of three participants each. After the first block, the project team reviewed the results and decided on any necessary changes in the measure. The process was repeated in blocks until a consensus was reached among block participants that the measure was readable and relevant, with no additional revisions required.

## Results

### Concept elicitation

#### Sample description

Interviews with seven experts were conducted in the US (*n* = 4) and Spain (*n* = 3). Six experts were clinical/medical experts in achondroplasia, and one expert was a leader in an achondroplasia advocacy group. Clinical/medical experts had a range of different specialties, including clinical/medical genetics (*n* = 3), pediatrics (*n* = 1), primary care (*n* = 1), skeletal dysplasias (*n* = 1), orthopedic surgery (*n* = 1), traumatology (*n* = 1), and clinical psychology (*n* = 1). Clinical experts had an average of 23 years of experience treating patients with achondroplasia (range 5–40 years). Experts worked in academic hospitals (33%, *n* = 2), public/other hospitals (33%, *n* = 2), a clinic (17%, *n* = 1), or were recently retired (17%, *n* = 1).

Thirty-six parents of children aged 2 to < 12 years with achondroplasia participated in individual/focus group interviews. Parent demographic characteristics are given in Table 1. Demographic and health characteristics for the children of parent participants are shown in Table 2.

**Table 1:** Parent concept elicitation participant demographic characteristics

	Spain (*n* = 11)	US (*n* = 25)	Total (*n* = 36)
Age, mean(SD)	40.4(3.1)	42.0(7.6)	41.5(6.6)
(Range)	(35–43)	(32–68)	(32–68)
Relationship to child, *n*(%)			
Mother	8(73)	23(92)	31(86)
Father	3(27)	2(8)	5(14)
Marital status, *n*(%)			
Single	2(18)	0	2(6)
Married	6(55)	23(92)	29(81)
Partnered	3(27)	0	3(8)
Divorced	0	2(8)	2(6)
Race/ethnicity, *n*(%)^a^			
Black/African American	–	3(12)	–
Latino/Hispanic	–	1(4)	–
White/Caucasian	–	23(92)	–
Education, *n*(%)			
Less than high school	2(18)	1(4)	3(8)
High school or equivalent	4(36)	2(8)	6(17)
College degree	5(46)	12(48)	17(47)
Post-graduate school	0	10(40)	10(28)
Work status, *n*(%)			
Full-time	6(55)	10(40)	16(44)
Part-time	3(27)	3(12)	6(17)
Student	0	2(8)	2(6)
Retired	0	1(4)	1(3)
Not working (other)	2(18)	9(36)	11(31)
Household income, *n*(%)^b^			
< 20,000	3(27)	1(4)	–
20,001 to 40,000	5(46)	2(8)	–
40,001 to 60,000	1(9)	1(4)	–
60,001 to 80,000	1(9)	3(12)	–
80,001 to 100,000	0	1(4)	–
> 100,000	0	15(60)	–
Decline to answer	1(9)	2(8)	–

Percentages may not add to 100 due to rounding

*SD* standard deviation

^a^US only; response categories are not mutually exclusive, so percentages do not add to 100

^b^Total household income was reported in Euros (€) for participants in Spain and in US dollars ($) for US participants

**Table 2:** Demographic/health characteristics for the children of parent participants

	Spain (*n* = 11)	US (*n* = 25)	Total (*n* = 36)
Child age, *n*(%)			
2 to < 5 years	5(46)	6(24)	11(31)
5 to < 9 years	4(36)	9(36)	13(36)
9 to < 12 years	2(18)	10(40)	12(33)
Child gender, *n*(%)			
Female	7(64)	12(48)	19(53)
Male	4(36)	13(52)	17(47)
Child’s race/ethnicity, *n*(%)^a^			
Asian-American	–	2(8)	–
Black/African-American	–	4(16)	–
Latino/Hispanic	–	2(8)	–
White/Caucasian	–	20(80)	–
Age/time diagnosed with achondroplasia, *n*(%)			
In utero	9(82)	12(48)	21(58)
At birth	1(9)	4(16)	5(14)
< 2 months of age	1(9)	2(8)	3(8)
2–6 months of age	0	5(20)	5(14)
Unknown (adopted)	0	2(8)	2(6)
Child has parent(s) with achondroplasia, *n*(%) yes	0	7(28)	7(19)
Health status (parent-reported), *n*(%)			
Excellent	3(27)	9(36)	12(33)
Very good	3(27)	11(44)	14(39)
Good	3(27)	4(16)	7(19)
Fair	2(18)	1(4)	3(8)
Height (cm)			
Mean(SD)	89.5(10.4)	93.2(15.2)	92.1(13.9)
(Range)	(75.0–104.0)	(63.5–121.0)	(63.5–121.0)
Weight (kg)			
Mean(SD)	17.3(5.6)	20.4(8.4)	19.4(7.7)
(Range)	(10.0–28.0)	(8.3–43.9)	(8.3–43.9)
BMI			
Mean(SD)	18.9(4.2)	21.4(7.0)	20.6(6.3)
(Range)	(13.3–27.5)	(12.6–44.7)	(12.6–44.7)
Planned limb lengthening surgery for the future, *n*(%) yes	5(46)	0	5(14)

Percentages may not add to 100 because of rounding

*SD* standard deviation; *BMI* body mass index

^a^US only; response categories are not mutually exclusive, so percentages do not add to 100

#### Expert interviews

Experts highlighted a range of issues and impacts affecting parents of children (< 18 years) with achondroplasia, including caretaking responsibilities, impacts on parent emotional and social well-being, impacts on parent work, and family impacts (Table 3). It should be noted that expert reports reflect impacts on parents of children with achondroplasia < 18 years, as experts were not asked questions specific to parents of children aged 2 to < 12 years. Although experts discussed some differences in parent impact by child age, it was not possible to report issues/impacts only affecting parents of children aged 2 to < 12 years, as experts tended to speak more generally about child age.

**Table 3:** Impacts on parent caretaking responsibilities

*n*, % reporting impact/issue	Parent reports by child age								Experts (*n* = 7)^a^	
	2 to < 5 years (*n* = 11)		5 to < 9 years (*n* = 13)		9 to < 12 years (*n* = 12)		Total (*n* = 36)			
Managing child's medical care/treatment	11	100%	12	92%	10	83%	33	92%	6	86%
Help with self-care	8	73%	10	77%	6	50%	24	67%	2	29%
Advocating for child	4	36%	10	77%	9	75%	23	64%	5	71%
General advocacy	3	27%	9	69%	6	50%	18	50%	5	71%
Educating others	1	9%	7	54%	6	50%	14	39%	2	29%
Assisting child with tasks (e.g., reaching objects)	8	73%	6	46%	6	50%	20	56%	2	29%
Support/guidance (e.g., for living with/managing achondroplasia)	4	36%	5	38%	8	67%	17	47%	6	86%
Monitoring child (e.g., for safety)	8	73%	6	46%	3	25%	17	47%	2	29%
Obtaining adaptations for child	2	18%	6	46%	1	8%	9	25%	3	43%
Finding childcare/school	4	36%	3	23%	0	0%	7	19%	1	14%

^a^ Expert reports reflect impacts on parents of children with achondroplasia aged < 18 years

Experts discussed ten caretaking responsibilities that parents of children with achondroplasia have, which are shown below (Table 3). The most frequently mentioned caretaking responsibilities included managing child’s medical care/treatment (86%, *n* = 6), providing support/guidance to child (e.g., for living with/managing achondroplasia; 86%, *n* = 6), and advocating for child (e.g., for accommodations, educating others; 71%, *n* = 5). Other caretaking responsibilities reported included obtaining adaptations for child (43%, *n* = 3), helping child with self-care (29%, *n* = 2), assisting child with tasks (e.g., reaching objects; 29%, *n* = 2), and monitoring child (e.g., for safety; 29%, *n* = 2).

Expert reports of impacts on parents’ emotional well-being associated with having a child with achondroplasia are given in Table 4. The most frequent emotional impacts on parents mentioned by experts included feeling worried/concerned (86%, *n* = 6), experiencing a period of initial shock/grief following child’s diagnosis (86%, *n* = 6), having increased knowledge of achondroplasia (71%, *n* = 5), and denial (e.g., difficulty accepting condition; 57%, *n* = 4), specific worry about child’s physical health (43%, *n* = 3), feeling stressed/overwhelmed (43%, *n* = 3), acceptance of child’s condition (43%, *n* = 3), being protective of child (43%, *n* = 3), intense focus on child (43%, *n* = 3), and feeling guilty (43%, *n* = 3). Experts did not report impacts on parents’ physical well-being due to having a child with achondroplasia.

**Table 4:** Impacts on parent emotional and physical well-being

*n*, % reporting impact	Parent reports by child age								Experts (*n* = 7)^a^	
	2 to < 5 years (*n* = 11)		5 to < 9 years (*n* = 13)		9 to < 12 years (*n* = 12)		Total (*n* = 36)			
Impacts on parent emotional well-being	11	100%	13	100%	12	100%	36	100%	7	100%
Worried/concerned	10	91%	12	92%	10	83%	32	89%	6	86%
Worry about the future	6	55%	12	92%	9	75%	27	75%	1	14%
Worry about child’s physical health	6	55%	9	69%	9	75%	24	67%	3	43%
Safety concerns	7	64%	6	46%	5	42%	18	50%	2	29%
Worry about child’s social well-being	3	27%	7	54%	5	42%	15	42%	1	14%
Concern about child’s ability to function independently	4	36%	6	46%	2	17%	12	33%	2	29%
Worry about child’s emotional well-being	0	0%	8	62%	3	25%	11	31%	0	0%
Stressed/overwhelmed	6	55%	4	31%	6	50%	16	44%	3	43%
Initial shock/grief	5	45%	7	54%	3	25%	15	42%	6	86%
Putting things in perspective	2	18%	5	38%	4	33%	11	31%	1	14%
Happy	4	36%	4	31%	2	17%	10	28%	0	0%
Acceptance	3	27%	2	15%	3	25%	8	22%	3	43%
Positive	3	27%	2	15%	3	25%	8	22%	0	0%
Fortunate/lucky	2	18%	3	23%	2	17%	7	19%	0	0%
Increased knowledge	2	18%	4	31%	1	8%	7	19%	5	71%
Proud	3	27%	2	15%	2	17%	7	19%	1	14%
Normal	2	18%	1	8%	3	25%	6	17%	1	14%
Protective of child	2	18%	3	23%	1	8%	6	17%	3	43%
Depressed/sad	3	27%	2	15%	0	0%	5	14%	2	29%
Empathy/understanding	0	0%	3	23%	2	17%	5	14%	0	0%
Overcoming challenges	0	0%	3	23%	1	8%	4	11%	1	14%
Hurt/bother	1	9%	2	15%	0	0%	3	8%	1	14%
Anxious/nervous	1	9%	1	8%	1	8%	3	8%	0	0%
Anger/frustration	0	0%	1	8%	2	17%	3	8%	1	14%
Grateful	1	9%	1	8%	1	8%	3	8%	0	0%
Strength/resilience	1	9%	1	8%	1	8%	3	8%	0	0%
Intense focus on child	1	9%	0	0%	1	8%	2	6%	3	43%
Self-conscious	0	0%	1	8%	1	8%	2	6%	1	14%
Alone/lonely	1	9%	0	0%	0	0%	1	3%	0	0%
Patience	0	0%	0	0%	1	8%	1	3%	0	0%
Confused	0	0%	0	0%	0	0%	0	0%	1	14%
Denial	0	0%	0	0%	0	0%	0	0%	4	57%
Guilty	0	0%	0	0%	0	0%	0	0%	3	43%
Impacts on parent physical well-being	5	45%	2	15%	3	25%	10	28%	0	0%
Being tired/sleep deprived	5	45%	1	8%	2	17%	8	22%	0	0%
General health	4	36%	1	8%	2	17%	7	19%	0	0%

^a^ Expert reports reflect impacts on parents of children with achondroplasia aged < 18 years

Limited impacts on parents’ social well-being related to having a child with achondroplasia were noted by experts (Table 5). The most often mentioned impact was parents having support/friendships through a community of people with dwarfism (29%, *n* = 2), followed by limiting social activities (14%, *n* = 1), and experiencing stigma/ignorance (14%, *n* = 1).

**Table 5:** Impacts on parent social well-being

*n*, % reporting impact/issue	Parent reports by child age								Experts (*n* = 7)^a^	
	2 to < 5 years (*n* = 11)		5 to < 9 years (*n* = 13)		9 to < 12 years (*n* = 12)		Total (*n* = 36)			
Support and friendships through a community of people with dwarfism	7	64%	9	69%	9	75%	25	69%	2	29%
Limit social/other activities	4	36%	3	23%	3	25%	10	28%	1	14%
Support from family/friends	4	36%	3	23%	3	25%	10	28%	0	0%
Strained relationships	2	18%	0	0%	0	0%	2	6%	0	0%
Stigma/ignorance	0	0%	0	0%	1	8%	1	3%	1	14%

^a^ Expert reports reflect impacts on parents of children with achondroplasia aged < 18 years

Experts also discussed the impacts of having a child with achondroplasia on families, as shown in Table 6. The most frequently mentioned impact was strain in the family (e.g., family stress, having less time; 86%, *n* = 6). Other impacts included family travel or vacations (e.g., prioritizing trips to advocacy organization meetings/events; 29%, *n* = 2) and limiting/adapting family activities (29%, *n* = 2). Impacts on siblings included strain on siblings (e.g., siblings receiving less time/attention from parents; 43%, *n* = 3) and caretaking responsibilities for their sibling with achondroplasia (29%, *n* = 2).

**Table 6:** Impacts on families

n, % reporting impact/issue	Parent reports by child age								Experts (*n* = 7)^a^	
	2 to < 5 years (*n* = 11)		5 to < 9 years (*n* = 13)		9 to < 12 years (*n* = 12)		Total (*n* = 36)			
Impacts on families										
Strain on family	5	45%	8	62%	7	58%	20	56%	6	86%
Family travel or vacations	5	45%	6	46%	8	67%	19	53%	2	29%
Limit/adapt family activities	5	45%	8	62%	2	17%	15	42%	2	29%
Increased family closeness	0	0%	3	23%	3	25%	6	17%	0	0%
Impacts on siblings										
Strain on siblings	4	36%	5	38%	4	33%	13	36%	3	43%
Caretaking responsibilities	2	18%	5	38%	4	33%	11	31%	2	29%
Increased empathy	0	0%	2	15%	3	25%	5	14%	1	14%
Other sibling impacts	1	9%	2	15%	1	8%	4	11%	1	14%

^a^ Expert reports reflect impacts on parents of children with achondroplasia aged < 18 years

Further, experts noted impacts on parent employment/work due to having a child with achondroplasia (Table 7). Four experts (57%, *n* = 4) discussed impacts on parent work, and the most often mentioned impacts were missed work time (43%, *n* = 3) and discontinued work to care for child (29%, *n* = 2).

**Table 7:** Impacts on parent employment/work

*n*, % reporting impact/issue	Parent reports by child age								Experts (*n* = 7)^a^	
	2 to < 5 years (*n* = 11)		5 to < 9 years (*n* = 13)		9 to < 12 years (*n* = 12)		Total (*n* = 36)			
Work/productivity issues	8	73%	11	85%	9	75%	28	78%	4	57%
Missed work time	4	36%	6	46%	8	67%	18	50%	3	43%
Changed work schedule	3	27%	2	15%	2	17%	7	19%	0	0%
Discontinued work	2	18%	3	23%	0	0%	5	14%	2	29%
Past time out of workforce	0	0%	1	8%	1	8%	2	6%	0	0%
Reduced work hours	0	0%	1	8%	1	8%	2	6%	1	14%
Difficult work/life balance	0	0%	0	0%	1	8%	1	3%	0	0%
Limited work opportunities	0	0%	1	8%	0	0%	1	3%	0	0%
Changed job/career	1	9%	0	0%	0	0%	1	3%	0	0%

^a^ Expert reports reflect impacts on parents of children with achondroplasia aged < 18 years

#### Parent interviews

In total, 175 concepts related to the physical signs/symptoms of achondroplasia in children (35 concepts) and the impacts of achondroplasia on children, parents, and families (140 concepts) were identified and coded in the parent interviews/focus group. Thematic saturation was assessed to ensure that the sample size was adequate for all relevant topics to be covered in the interviews. A saturation grid was created for the 36 parent participants in the chronological order in which the interviews/focus group occurred. After the 6th participant, 75% of concepts had been discussed. Following the 23rd participant, saturation was considered reached, with 96% of concepts covered.

Participants described eight caretaking responsibilities related to having a child with achondroplasia. The most frequently mentioned were managing child’s medical care/treatment (92%, *n* = 33), helping child with self-care (e.g., toileting, bathing; 67%, *n* = 24), advocating for child (64%, *n* = 23), and assisting child with tasks (e.g., reaching objects for child; 56%, *n* = 20). Other often mentioned caretaking responsibilities included providing child with support/guidance for living with/managing achondroplasia (47%, *n* = 17), monitoring child (e.g., for safety; 47%, *n* = 17), and obtaining adaptations for child (25%, *n* = 9).

Parents also discussed 29 emotions experienced in relation to their child’s achondroplasia, as well as coping strategies parents used for managing their emotions. The most often mentioned emotional impact was feeling worried or concerned (89%, *n* = 32). Parents most frequently worried about the future (75%, *n* = 27), followed by worry about their child’s physical health (67%, *n* = 24), safety concerns for their child (50%, *n* = 18), worry about child’s social well-being (42%, *n* = 15), concern about child’s ability to function independently (33%, *n* = 12), and worry about child’s emotional well-being (31%, *n* = 11). Parents also frequently discussed feeling stressed/overwhelmed (44%, *n* = 16) and experiencing an initial period of shock/grief following their child’s diagnosis (42%, *n* = 15). In addition to more difficult emotions, a number of parents expressed positive emotions in relation to their child’s achondroplasia, including feeling happy (28%, *n* = 10), positive (22%, *n* = 8), fortunate/lucky (19%, *n* = 7), proud (19%, *n* = 7), and having increased knowledge of achondroplasia (19%, *n* = 7). Moreover, many parents discussed coping strategies, including putting things in perspective (31%, *n* = 11) and acceptance (22%, *n* = 8). Fewer parents reported impacts on their physical well-being. Impacts on parent physical health included being tired/sleep deprived (22%, *n* = 8) and general/other health impacts (19%, *n* = 7).

Parents reported five impacts on their social well-being related to having a child with achondroplasia. The most often mentioned impact was having social connections, support, and/or friendships through a community of people with dwarfism, typically through an advocacy organization (69%, *n* = 25). Many parents described these connections as important sources of education and support. Other impacts included parents limiting their social/other activities (28%, *n* = 10) and having a support network of family/friends (28%, *n* = 10).

The most frequently mentioned impacts on families included strain on the family (56%, *n* = 20), family travel/vacations (e.g., prioritizing advocacy organization meetings/events for family vacations; 53%, *n* = 19), and limiting/adapting family activities to accommodate child with achondroplasia (42%, *n* = 15). Some parents reported increased family closeness due to having a child with achondroplasia (17%, *n* = 6). Parents also discussed impacts on siblings, which most frequently included strain on siblings (e.g., sibling without achondroplasia receiving less time or attention; 36%, *n* = 13) and caretaking responsibilities for their sibling with achondroplasia (31%, *n* = 11).

Most parents (78%, *n* = 28) indicated that having a child with achondroplasia affected their employment or work. The most often mentioned impacts on work were missed work time (e.g., coming in late, leaving early, or missing a full day of work; 50%, *n* = 18), needing to change their work schedule (e.g., working at a different time/shift; 19%, *n* = 7), and discontinuing work to care for child (14%, *n* = 5).

#### Preliminary theoretical model

**Fig. 1:**
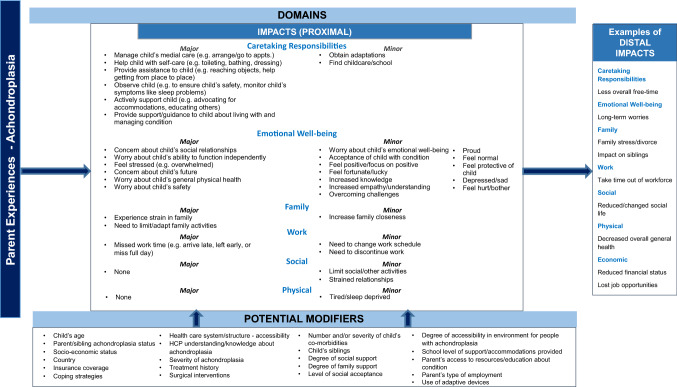
Preliminary theoretical model for parent experiences/impacts of having a child with achondroplasia (ages 2 to < 12 years)

Based on the qualitative analysis of the CE interviews, a preliminary theoretical model of the impacts of having a child with achondroplasia on parents was developed (Fig. 1). The theoretical model illustrates a range of impacts on parents in the areas of caretaking, emotional well-being, family, work, social well-being, and physical well-being. Moreover, the model identifies major vs. minor impacts, proximal (immediate) impacts, and distal (longer-term) impacts. Additionally, the model suggests modifying factors that may affect the impacts on parents, such as child age, health insurance coverage, and parent coping strategies.

The final criteria that the research team agreed upon to designate parent impacts as major included the following:

1. Endorsement by at least 30% of parents in at least two of the three child age groups analyzed; or an endorsement by 25–29% of parents in at least two of the three age groups if conceptually important
2. Potentially responsive to a new treatment for children
3. Would be considered bothersome, limiting, or difficult
4. Impacts must be temporally proximal

For impacts that were not considered major, the criteria agreed upon for identifying a minor impact were as follows:

1. Endorsement by at least 10% of parents in at least one of the three child age groups analyzed
2. Impacts must be temporally proximal

### Item generation

To help ensure that the parent impact measure would be responsive to change, without floor/ceiling effects, only major impacts that were bothersome, limiting, or difficult and could potentially improve with a new treatment for children were considered for measure items. Using the criteria outlined above, a draft of the preliminary APEM measure was generated. Based on the translatability assessment report, minor edits were made to some of the wording of items to avoid potential difficulties translating the APEM into other languages.

#### Cognitive debriefing

Sixteen parents participated in individual CD telephone interviews. Demographic characteristics of parent participants and the demographic/health characteristics of their children are presented in Table 8.

**Table 8:** Cognitive debriefing parent participant and child demographic characteristics

Parent participant demographics		Child demographic/health characteristics	
Parent age, mean(SD)	39.1(6.4)	Child age,* n*(%)	
(Range)	(31–55)	2 to < 5 years	4(25)
Relationship to child, *n*(%)		5 to < 9 years	6(38)
Mother	16(100)	9 to < 12 years	6(38)
Parent has achondroplasia, *n*(%) yes	2(12.5)	Child gender,* n*(%)	
Marital status, *n*(%)		Female	12(75)
Single	1(6)	Male	4(25)
Married	14(88)	Age/time diagnosed with achondroplasia,* n*(%)	
No response	1(6)	In utero	5(31)
Parent race/ethnicity, *n*(%)^a^		At birth	1(6)
Asian-American	1(6)	< 2 months of age	2(13)
Latino/Hispanic	1(6)	2–6 months of age	5(31)
White/Caucasian	14(88)	> 6 months of age	1(6)
Parent education, *n*(%)		Unknown (adopted)	2(13)
High school or equivalent	2(13)	Child’s race/ethnicity,* n*(%)^a^	
Vocational/technical school	1(6)	Asian-American	4(25)
College degree	9(56)	Latino/Hispanic	1(6)
Post-graduate school	4(25)	White/Caucasian	11(69)
Parent work status, *n*(%)		Child health status (parent-reported), *n*(%)	
Full-time	7(44)	Excellent	8(50)
Part-time	4(25)	Very good	7(44)
Retired/student	0	Good	0
Not working (other)	5(31)	Fair	1(6)
Household income, *n*(%)		Child height (cm)	
< $40,000	0	Mean(SD)	90.8(12.0)
$40,001 to $60,000	1(6)	(Range)	(66.0–106.7)
$60,001 to $80,000	1(6)	Child weight (kg)	
$80,001 to $100,000	6(38)	Mean(SD)	18.3(5.8)
> $100,000	6(38)	(Range)	(10.9–31.7)
Decline to answer	2(13)	BMI	
		Mean(SD)	19.8(4.5)
		(Range)	(14.8–32.0)

*n* = 16. Percentages may not add to 100 due to rounding

*SD* standard deviation, *BMI* body mass index

^a^Response categories are not mutually exclusive

Five blocks of parent CD interviews were needed to revise the measure and items to improve readability and relevance. A sixth, confirmatory block of interviews was conducted with three parents (two block one participants and one new participant) to affirm readability and relevance of the measure.

#### Preliminary measure

**Fig. 2:**
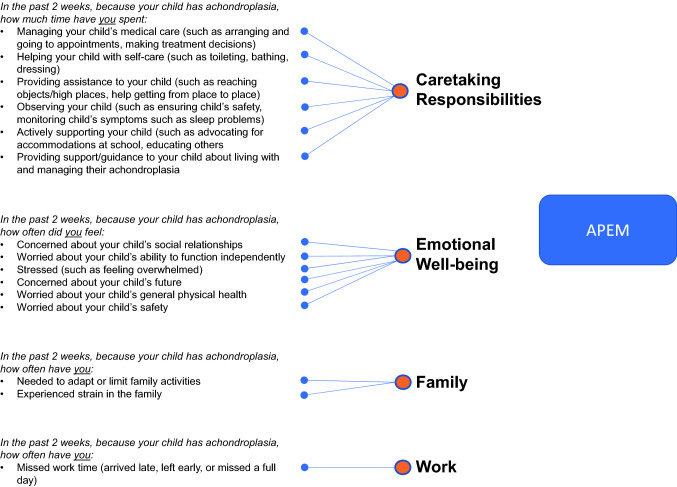
Preliminary conceptual framework of APEM

Following the CD interviews, a validation-ready version of the APEM was created. The APEM reflects four domains of parental impacts, including Caretaking Responsibilities, Emotional Well-being, Family, and Work. The preliminary conceptual framework for the APEM is shown in Fig. 2. The APEM consists of 15 items using three question stems with corresponding response options. All items include a five-point, Likert-type response scale, which ensured that meaningful distinctions could be made between response options, while minimizing the cognitive burden for respondents [24]. Items measuring frequency or “how often” have response options ranging from “Never” to “Very often/always.” For items measuring the amount of time, the response scale ranges from “None” to “A great deal.” To minimize recall bias, the recall period used for all items in the measure was the previous two weeks. The APEM is intended for parents/guardians of children aged 2 to < 12 years with achondroplasia.

## Discussion

The purpose of the study was to conduct a parent-centered, qualitative investigation of how having a child with achondroplasia affects parents’ daily lives and well-being. CE interviews were conducted with experts and parents to better understand the experiences of parents of children with achondroplasia aged 2 to < 12 years. A qualitative analysis of the CE interviews, based on an adapted grounded theory approach, was conducted, and a preliminary theoretical model depicting the impacts of having a child with achondroplasia on parents was developed. The qualitative analysis and theoretical model informed the development of the APEM, a parent-reported experience measure of the impacts of having a child with achondroplasia aged 2 to < 12 years on parent daily life and well-being. CD interviews conducted for the APEM confirmed parent understanding and item relevance. A psychometric study of the APEM is needed to validate the measure. To our knowledge, this is the first parent-centered and condition-specific measure of the broad spectrum of impacts of having a child with achondroplasia on parents, developed in accordance with FDA guidelines and best research practices for PRO development [19–22].

The findings suggest that having a child with achondroplasia may impact parents in a range of different ways, including caretaking responsibilities, emotional well-being, family life, social well-being, and work. The study is consistent with previous research, which has shown that parents of children with achondroplasia have greater caretaking responsibilities, such as helping child with self-care, compared to parents of typically developing children [14]. The study also confirms prior research indicating that having a child with achondroplasia affects parents’ emotional well-being, but has less of an impact on parents’ physical well-being [13, 17]. Parents frequently expressed worries or concerns regarding their child with achondroplasia. The most often mentioned family impacts included family strain, limiting/adapting family activities, and adapting family travel/vacation plans. It is notable that a majority of parents discussed the social connections and support they received through a community of people with dwarfism, primarily through advocacy organizations. The most frequent impact on parent work was missed work time.

In addition, the qualitative findings provide evidence to support the content validity of the APEM. Given the new treatments currently in development for children with achondroplasia, it is crucial for researchers and clinicians to understand the impacts of having a child with achondroplasia on parents and families, in addition to impacts on children’s well-being. Following psychometric validation, the APEM will be a useful assessment of the impacts of having a child with achondroplasia on parents for use in future research, including the evaluation of new treatments for children.

The study findings also have important implications for clinical practice and may add to current clinical guidelines for the management of achondroplasia in children [8]. The large differences in some reported impacts between parents and experts suggest that clinicians may be less aware of some parental impacts and highlight the importance of assessing impacts from the parent perspective, in addition to clinical outcome assessments. For instance, 67% of parents discussed helping their child with self-care, while only 29% of experts mentioned help with self-care. Likewise, while 75% of parents reported worry about the future, only 14% of experts discussed this. Recognizing the different ways in which having a child with achondroplasia affects parents and families may improve clinician and parent communication and allow clinicians to provide parents with education and support for the challenges they may face in the areas of caretaking, emotional well-being, family life, and work. The validated APEM may serve as a useful assessment tool for clinicians to gauge impacts on the parents of children in their care and develop treatment plans that consider parents and families.

Study limitations should be considered when interpreting results. Due to the study sample size, statistical significance tests of sub-group differences in results were not conducted. Therefore, any reported percentage differences may not reflect actual population differences, and results should be interpreted with caution. Although a multi-pronged recruitment strategy was used, most participants were recruited through dwarfism advocacy organizations, which may increase the risk of selection bias, as parents who are involved with advocacy organizations may differ from those who are not. Additionally, given that the CE interviews were conducted in the US and Spain and CD interviews were conducted in the US, the results may not be generalizable to other countries with differing cultures and healthcare systems. Future research should investigate potential cross-country differences in parents’ experiences. When the validated APEM is translated into other languages, additional CD interviews will be conducted in each country in which the measure is to be used as part of the cultural validation process.

The APEM was designed to assess impacts on parents of children with achondroplasia of diverse ages. This relatively broad child age target ensures that the APEM would be relevant across children’s age span and a useful assessment tool in clinical trials and longitudinal research studies, which may last many years. Due to the relatively broad child age span, there may be a small number of impacts that are relevant and important to parents of older/younger children that were not included in the measure. For instance, 45% of parents of children aged 2 to < 5 years reported feeling tired/sleep deprived, while few parents of older children reported this issue. Some of the more specific impacts/concepts that were not included as items were included in higher-order concepts covered. For instance, although the APEM does not have a specific item for worry about child’s emotional well-being, parents in the study who indicated this specific worry also expressed other, more general worries that were included in the APEM, such as worry about the future. Additionally, some items that were included in the APEM may be less relevant for parents in one child age group compared to another. For example, only 25% of parents of older children (9 to < 12 years) reported monitoring their children (e.g., for safety), compared to 73% of parents of young children (2 to < 5 years). Nevertheless, all impacts in the APEM were reported by parents in all three child age groups. Future research might explore the impacts of having an infant/young child aged < 2 years or an adolescent with achondroplasia on parents.

## Conclusion

The findings highlight the importance of considering how achondroplasia impacts parents’ well-being, in addition to the well-being of affected children. This study has shown that having a child with achondroplasia affects parents in the areas of caretaking responsibilities, emotional well-being, family life, and work. The qualitative analysis also provides evidence of the content validity of the APEM. Following psychometric validation, the APEM will be a useful tool for researchers and clinicians to assess the broad impacts of having a child with achondroplasia on parents and families and to evaluate newly developed treatments for children with achondroplasia.

## Data Availability

The data for the research presented in the publication may be available from the corresponding author on reasonable request.
