# Correction to: Assessing the impacts of having a child with achondroplasia on parent well-being

**DOI:** 10.1007/s11136-020-02659-3

**Published:** 2020-10-30

**Authors:** Kathryn M. Pfeiffer, Meryl Brod, Alden Smith, Jill Gianettoni, Dorthe Viuff, Sho Ota, R. Will Charlton

**Affiliations:** 1 The Brod Group, 219 Julia Ave., Mill Valley, CA 94941 USA; 2 Ascendis Pharma, Inc, Palo Alto, CA USA; 3 Ascendis Pharma, A/S, Hellerup, Denmark

## Correction to: Quality of Life Research 10.1007/s11136-020-02594-3

In the original publication, the summary statistics for child BMI in Tables 2 and 8 were incorrect. The correct version of Tables 2 and 8 (with updated summary statistics) are provided in this correction.

**Table 2:** Demographic/health characteristics for the children of parent participants

	Spain (*n* = 11)	US (*n* = 25)	Total (*n* = 36)
Child age, *n*(%)			
2 to < 5 years	5(46)	6(24)	11(31)
5 to < 9 years	4(36)	9(36)	13(36)
9 to < 12 years	2(18)	10(40)	12(33)
Child gender, *n*(%)			
Female	7(64)	12(48)	19(53)
Male	4(36)	13(52)	17(47)
Child’s race/ethnicity, *n*(%)^a^			
Asian-American	–	2(8)	–
Black/African-American	–	4(16)	–
Latino/Hispanic	–	2(8)	–
White/Caucasian	–	20(80)	–
Age/time diagnosed with achondroplasia, *n*(%)			
In utero	9(82)	12(48)	21(58)
At birth	1(9)	4(16)	5(14)
< 2 months of age	1(9)	2(8)	3(8)
2–6 months of age	0	5(20)	5(14)
Unknown (adopted)	0	2(8)	2(6)
Child has parent(s) with achondroplasia, *n*(%) yes	0	7(28)	7(19)
Health status (parent-reported), *n*(%)			
Excellent	3(27)	9(36)	12(33)
Very good	3(27)	11(44)	14(39)
Good	3(27)	4(16)	7(19)
Fair	2(18)	1(4)	3(8)
Height (cm)			
Mean(SD)	89.5(10.4)	93.2(15.2)	92.1(13.9)
(Range)	(75.0–104.0)	(63.5–121.0)	(63.5–121.0)
Weight (kg)			
Mean(SD)	17.3(5.6)	20.4(8.4)	19.4(7.7)
(Range)	(10.0–28.0)	(8.3–43.9)	(8.3–43.9)
BMI			
Mean(SD)	21.0(3.0)	23.0(6.5)	22.4(5.7)
(Range)	(16.7–26.9)	(18.1–45.4)	(16.7–45.4)
Planned limb lengthening surgery for the future, *n*(%) yes	5(46)	0	5(14)

Percentages may not add to 100 because of rounding

*SD* standard deviation; *BMI* body mass index

^a^US only; response categories are not mutually exclusive, so percentages do not add to 100

**Table 8:** Cognitive debriefing parent participant and child demographic characteristics

Parent participant demographics		Child demographic/health characteristics	
Parent age, mean(SD)	39.1(6.4)	Child age,* n*(%)	
(Range)	(31–55)	2 to < 5 years	4(25)
Relationship to child, *n*(%)		5 to < 9 years	6(38)
Mother	16(100)	9 to < 12 years	6(38)
Parent has achondroplasia, *n*(%) yes	2(12.5)	Child gender,* n*(%)	
Marital status, *n*(%)		Female	12(75)
Single	1(6)	Male	4(25)
Married	14(88)	Age/time diagnosed with achondroplasia,* n*(%)	
No response	1(6)	In utero	5(31)
Parent race/ethnicity, *n*(%)^a^		At birth	1(6)
Asian-American	1(6)	< 2 months of age	2(13)
Latino/Hispanic	1(6)	2–6 months of age	5(31)
White/Caucasian	14(88)	> 6 months of age	1(6)
Parent education, *n*(%)		Unknown (adopted)	2(13)
High school or equivalent	2(13)	Child’s race/ethnicity,* n*(%)^a^	
Vocational/technical school	1(6)	Asian-American	4(25)
College degree	9(56)	Latino/Hispanic	1(6)
Post-graduate school	4(25)	White/Caucasian	11(69)
Parent work status, *n*(%)		Child health status (parent-reported), *n*(%)	
Full-time	7(44)	Excellent	8(50)
Part-time	4(25)	Very good	7(44)
Retired/student	0	Good	0
Not working (other)	5(31)	Fair	1(6)
Household income, *n*(%)		Child height (cm)	
< $40,000	0	Mean(SD)	90.8(12.0)
$40,001 to $60,000	1(6)	(Range)	(66.0–106.7)
$60,001 to $80,000	1(6)	Child weight (kg)	
$80,001 to $100,000	6(38)	Mean(SD)	18.3(5.8)
> $100,000	6(38)	(Range)	(10.9–31.7)
Decline to answer	2(13)	BMI	
		Mean(SD)	21.8(3.8)
		(Range)	(16.3–32.4)

*n* = 16. Percentages may not add to 100 due to rounding

*SD* standard deviation, *BMI* body mass index

^a^Response categories are not mutually exclusive

